# DNA Origami-Templated Bimetallic Nanostar Assemblies for Ultra-Sensitive Detection of Dopamine

**DOI:** 10.3389/fchem.2021.772267

**Published:** 2021-12-23

**Authors:** Vishaldeep Kaur, Mridu Sharma, Tapasi Sen

**Affiliations:** Institute of Nano Science and Technology, Mohali, India

**Keywords:** DNA origami, plasmonic nanostructures, surface-enhanced Raman scattering, dopamine, Ag coated Au nanostar

## Abstract

The abundance of hotspots tuned via precise arrangement of coupled plasmonic nanostructures highly boost the surface-enhanced Raman scattering (SERS) signal enhancements, expanding their potential applicability to a diverse range of applications. Herein, nanoscale assembly of Ag coated Au nanostars in dimer and trimer configurations with tunable nanogap was achieved using programmable DNA origami technique. The resulting assemblies were then utilized for SERS-based ultra-sensitive detection of an important neurotransmitter, dopamine. The trimer assemblies were able to detect dopamine with picomolar sensitivity, and the assembled dimer structures achieved SERS sensitivity as low as 1 fM with a limit of detection of 0.225 fM. Overall, such coupled nanoarchitectures with superior plasmon tunability are promising to explore new avenues in biomedical diagnostic applications.

## Introduction

Early detection of alterations in the level of key neuromodulators in our body is of central importance for identification of most neurodegenerative disorders, such as Parkinson’s disease (Lotharius and Brundin, 2002), Alzheimer’s disease (Henstridge et al., 2019), schizophrenia (Breier et al., 1997), and Huntington’s disease (Chen et al., 2013). Dopamine, a significant catecholamine neurotransmitter is responsible for the control and regulation of various brain functions in mammals (Beninger, 1983; Klein et al., 2019). Thus, imbalances in dopamine level is a diagnostic indicator to neural dysfunctions that can aid in monitoring and control of many neurological diseases. Mostly, fluorescence (Zhao et al., 2016; Gao et al., 2017), colorimetric (Lee et al., 2012; Feng et al., 2013), ELISA-based assays (Kim et al., 2008; Nichkova et al., 2013), chromatography (Peterson et al., 2002; Wu et al., 2016), and electrochemical methods (Wu et al., 2012; Sajid et al., 2016) are used extensively for the detection of dopamine. Although progress in sensitive detection of dopamine is reported in some methods (Sansuk et al., 2013; Ban et al., 2015; Talemi et al., 2017), these methods still pose a significant challenge in their potential practical applicability due to time-consuming, complicated sample preparation techniques, and limited accuracy (Pradhan et al., 2014; Tang et al., 2015). Hence, development of highly sensitive detection techniques is required to design low-cost diagnostic platforms for dopamine detection.

Surface-enhanced Raman scattering (SERS), on the other hand, is a highly sensitive analytical technique that can provide specific molecular fingerprints of analytes utilizing high electric fields generated near metal nanostructures (Stiles et al., 2008; Cialla et al., 2012; Ding et al., 2017). In the past decade, SERS has broadened its scope in many applications including label-free chemical and biological sensing (Vo-Dinh, 1995; Bantz et al., 2011; Shvalya et al., 2020), biomedical applications (Cialla-May et al., 2017; Huang et al., 2019), and detection down to single molecule level (Lim et al., 2010; Chen et al., 2015; Almehmadi et al., 2019). The strength of SERS signal is highly dominated by the formation of hotspots in well-defined plasmonic architectures as the presence of random and discrete hotspots limit the practical applicability of SERS nanosensors. The dimer and trimer nanostructures are the simplest geometries to determine the impact of multiple hotspot formation in coupled plasmonic nanostructures. For example, size-specific gold (Au) nanoparticles (Pal et al., 2019), spherical Au@Ag NPs (Chen et al., 2010), and Au and Ag NPs (Sergiienko et al., 2017) were synthesized to explore the stoichiometry effect on SERS signal enhancement. Various metallic nanostructure-based SERS substrates, such as Au/Ag nanoclusters (Phung et al., 2018), self-assembled Au nanoparticles (An et al., 2011), Ag nanocubes (Lu et al., 2020), worm-like Ag clusters (Palanisamy et al., 2015), and spread spectrum SERS technique (Lee et al., 2021), were developed in the last few years as probes for sensitive dopamine detection. These strategies are, however, limited by the experimental challenges for the systematic arrangement of nanostructures with nanometer-scale precision. Therefore, design of inexpensive, precisely-arranged coupled nanostructures based SERS substrates is highly required to generate strongly localized electromagnetic fields.

The technique of DNA origami is effective to design self-assembled geometries with nanometer precision creating highly customized nano-assemblies consisting of metallic nanoparticles (Ding et al., 2010; Hung et al., 2010; Pal et al., 2010), quantum dots (Bui et al., 2010), and fluorophores (Schreiber et al., 2014). Over the past few years, the programmability of DNA origami structures has enabled the periodic arrangement of wide variety of anisotropic nanostructures, resulting in several orders of electromagnetic field enhancement (Lan et al., 2013; Liu et al., 2017; Tanwar et al., 2017; Zhan et al., 2018). More significantly, bimetallic Au–Ag nanostructures lead to extraordinary electromagnetic enhancements as they combine the plasmonic properties of both the metals in singular structure (Cui et al., 2006; Fan et al., 2013; Feng et al., 2019). Recently, our group demonstrated assembly of bimetallic Ag coated Au nanostars (Au@Ag NSs) on DNA origami for the ultrasensitive SERS based detection of a bacterial biomarker, pyocyanin (Kaur et al., 2021). In a very recent study, the designed bimetallic nanoantennas were utilized for the broadband SERS enhancement of single dye molecules emitting in different spectral region, and the specific and label-free detection of a single protein molecule placed in the junction of dimer nanoantenna (Tanwar et al., 2021).

Herein, bimetallic Au core-Ag shell nanostars (Au@Ag NSs) were assembled on dimerized DNA origami with controlled stoichiometry, and the utilization of such assemblies for SERS-based detection of important neurotransmitter, dopamine was demonstrated. These nanoantennas have the benefit of better plasmonic response of Ag along with sharp tips of Au NSs and tiny interparticle gap (core-core) resulting in huge electromagnetic enhancements. To explore the influence of multiple hotspot formation, Au@Ag NSs with variable stoichiometry, i.e., dimer and trimer structures were assembled on dimerized DNA origami. The designed trimer system was able to achieve label-free SERS detection of dopamine with picomolar range of concentration, while dimeric structures achieved ultra-sensitive detection in femtomolar range. These findings suggest that such high precision and tunable DNA origami-directed nanoantennas can act as suitable platforms for biomolecular recognition and can benefit the early diagnosis of many related diseases.

## Materials and Methods

### Materials

All the HPLC purified DNA oligonucleotides were purchased from Integrated DNA technologies (IDT) and used without further purification. M13mp18 single-stranded DNA was procured from New England Biolabs. Gold chloride trihydrate (HAuCl_4_.3H_2_O, purity ≥99.9%), trisodium citrate dihydrate (Na_3_C_6_H_5_O_7_), L-ascorbic acid, silver nitrate (AgNO_3_), dopamine.HCl, tris-(carboxyethyl) phosphine hydrochloride (TCEP.HCl), magnesium chloride hexahydrate (MgCl_2_.6H_2_O), 1 M potassium phosphate monobasic solution (KH_2_PO_4_), 1 M potassium phosphate dibasic solution (K_2_HPO_4_), L-tyrosine and sodium chloride (NaCl) were purchased from Sigma-Aldrich. Tween 20, ammonium hydroxide (NH_4_OH, 25%) and hydrochloric acid (HCl) were purchased from Merck. L-DOPA was procured from SRL, and tyramine was purchased from TCI Chemicals. 1× TE buffer was purchased from IDT and 50× TAE buffer solution was procured from Himedia and used without further purification. Sephacryl S-300 high resolution resin was purchased from GE Healthcare. All the glasswares used in the experiments were thoroughly cleaned with aqua regia, water, and MilliQ water before using. MilliQ water was used for all the experiments.

### Methods

#### Synthesis of Dimerized DNA Origami

The rectangular DNA origami monomer was prepared according to the method designed by Paul Rothemund (Rothemund, 2006). Briefly, m13mp18 scaffold was mixed with ∼200 short staples in 1× TAE buffer with 12.5 mM MgCl_2_ and annealed in a PCR thermocycler followed by slow cooling. The synthesized DNA origami monomer was then dimerized according to the method reported earlier (Tanwar et al., 2017). The monomer solutions were mixed with 24 branching staples (40-fold excess) in 50× TAE with 12.5 mM MgCl_2_ and kept for 24 h at room temperature followed by purification with Sephacryl S-300 HR resin.

#### Design of Ag Coated Au Nanostar Dimer and Trimer Structures on Dimerized DNA Origami

Au NSs were synthesized according to a previously reported modified protocol (Tanwar et al., 2017). To achieve Ag coating, 1 μl of 0.1 M AgNO_3_, 1 μl of 0.1 M ascorbic acid, and 1 μl of NH_4_OH solution were subsequently added to 1 ml solution of Au NSs as described earlier (Kaur et al., 2021). The washed Au@Ag NSs were then functionalized with 5′ thiol terminated DNA with sequence 5′-SH-CGTCGTATTCGATAGCTTAG-3′ using a previously described method (Kaur et al., 2021). Briefly, deprotected thiolated DNA was incubated with Au@Ag NSs overnight followed by slow salt addition until 750 mM final concentration. After keeping overnight, the solution was washed using PBS containing 100 mM NaCl buffer solution followed by redispersion in 0.5× TAE. To prepare Au@Ag NSs-DNA origami assemblies, DNA-conjugated Au@Ag NSs were mixed with dimerized DNA origami in 2:1 M ratio in 0.5× TAE with 300 mM NaCl and heated repeatedly from 20°C to 40°C in PCR thermocycler. The position of modified staples on each monomer origami was varied so as to form Au@Ag NSs dimer and trimer assemblies. A total of five and eight modified strands were extended at 3′ end on both origami monomers for the immobilization of Au@Ag NSs dimers and trimers, respectively.

#### Detection of Dopamine Using DNA Origami–Ag Coated Au Nanostar Dimer and Trimer Assemblies

The DNA origami–Au@Ag NS assemblies were first incubated on plasma-cleaned Si wafer after dilution with 10× TAE buffer having 12.5 mM MgCl_2_ for 2.5 h. The final concentration of Au@Ag NSs structures on Si substrate was found to be ∼1.32 nM calculated according to the method reported in our recently published report (Kaur et al., 2021). After incubation, the substrate was washed thoroughly using MQ water. A stock solution of 1 mM dopamine solution was prepared in MQ water and subsequently diluted to prepare the desired concentration. A 10 µl aliquot of the desired concentration of dopamine solution was drop-casted on Au@Ag NSs assemblies on Si substrate. The solution was left for drying followed by washing to record Raman measurements.

### Characterization

Ultraviolet-visible (UV-Vis) absorption spectra were recorded with a Shimadzu UV-2600 spectrophotometer. TEM imaging was done using a JEOL 2100 microscope at an accelerating voltage of 200 kV for Au@Ag NSs and 120 kV for DNA origami samples. AFM imaging was done using a Bruker Multimode 8 scanning probe microscope with TAP 150-Al-G cantilevers (Budget sensor). The Raman measurements were conducted using Renishaw inVia confocal Raman microscope equipped with a 514 nm laser using 2.5 mW power with 10 s accumulation time.

## Results and Discussion

### Design of Ag Coated Au Nanostar Dimer and Trimer Assemblies on Dimerized DNA Origami

**FIGURE 1:**
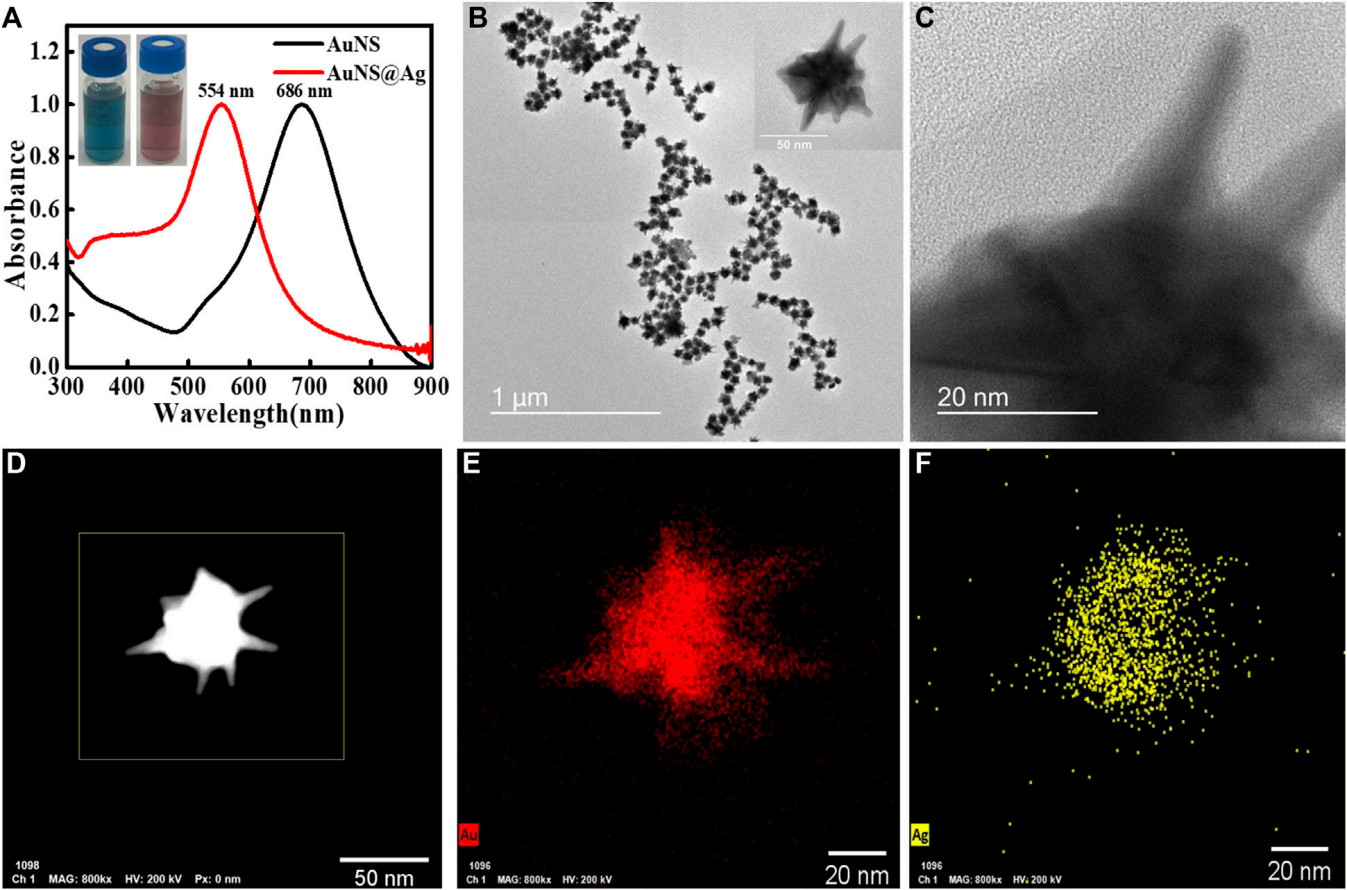
**(A)** Overlapping ultraviolet-visible (UV-Vis) spectra of Au nanostars (NSs) and Au@Ag NSs, **(B,C)** TEM and HRTEM images of Au@Ag NSs, **(D)** STEM HAADF image, and **(E,F)** corresponding elemental mapping of Au@Ag NS.

The synthetic strategy to prepare Au@Ag NSs was based on our recently reported method (Kaur et al., 2021). Ag coating on seed-mediated synthesized Au NSs was achieved by the subsequent addition of AgNO_3_, ascorbic acid, and NH_4_OH solution. The concentration of precursors was precisely controlled in order to achieve uniform coating on the nanostars while at the same time keeping the tips unaltered. Figure 1A shows that the LSPR of Au NSs blue shifted from 686 to 554 nm indicating the deposition of Ag on Au NSs. In addition to spectral shift, TEM images confirmed the coating of a thin layer of Ag on Au NSs (Figures 1B, C). From a statistical analysis of additional TEM images, the average size, i.e., tip–tip distance of Au@Ag NSs was calculated to be 60 ± 5 nm. From the HRTEM images statistical analysis, the thickness of Ag layer was estimated to be 2.4 ± 0.5 nm. The STEM-EDX elemental mapping further confirmed that thin Ag layer was getting deposited on the core as well as the tips of Au NSs (Figures 1D–F). Additionally, the EDX spectrum (Supplementary Figure S1) also revealed the presence of Au and Ag components in Au@Ag NSs.

**SCHEME 1:**
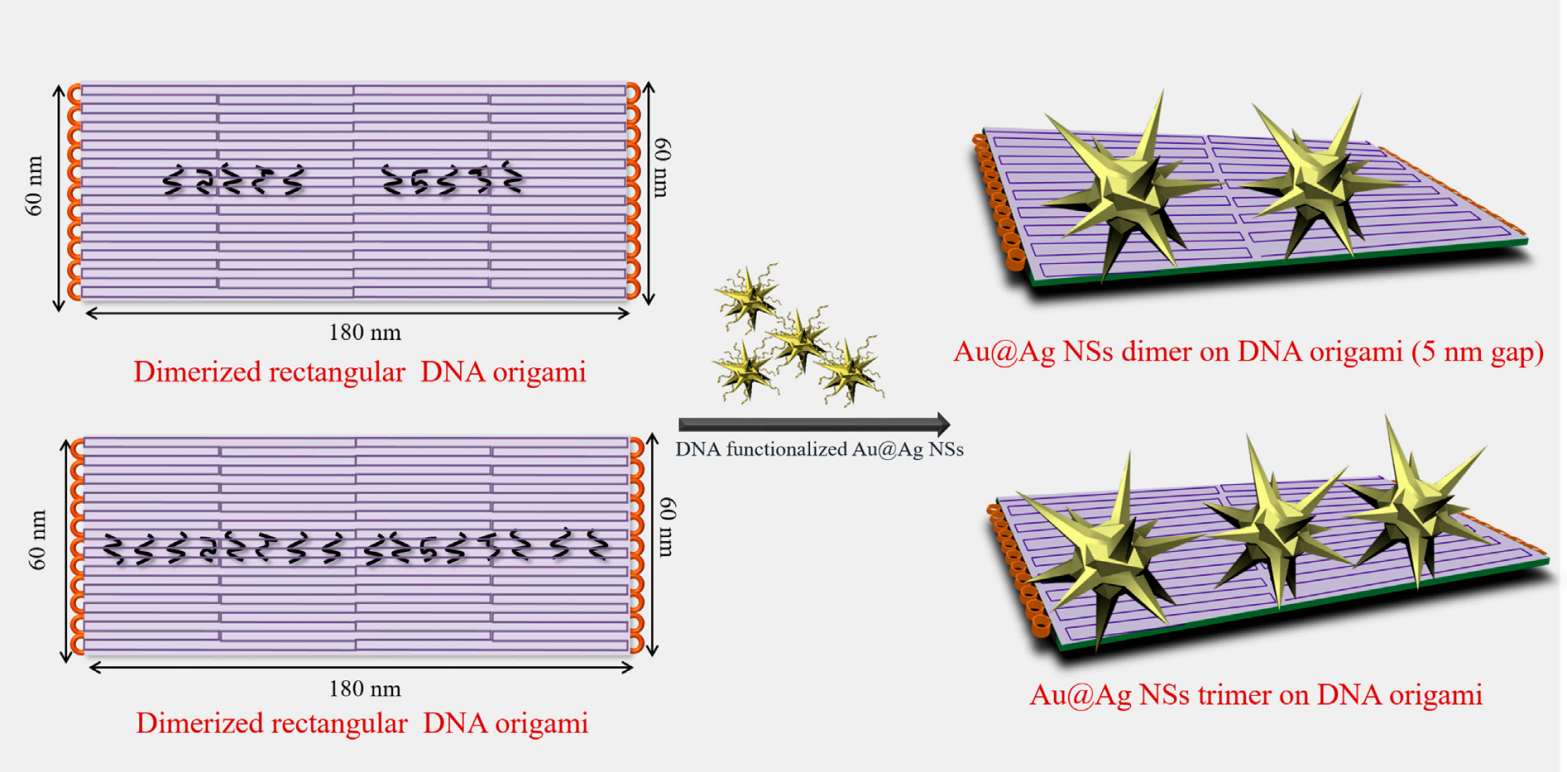
Schematic depiction of design of Au@Ag nanostars (NSs) dimer and trimer on dimerized DNA origami.

**FIGURE 2:**
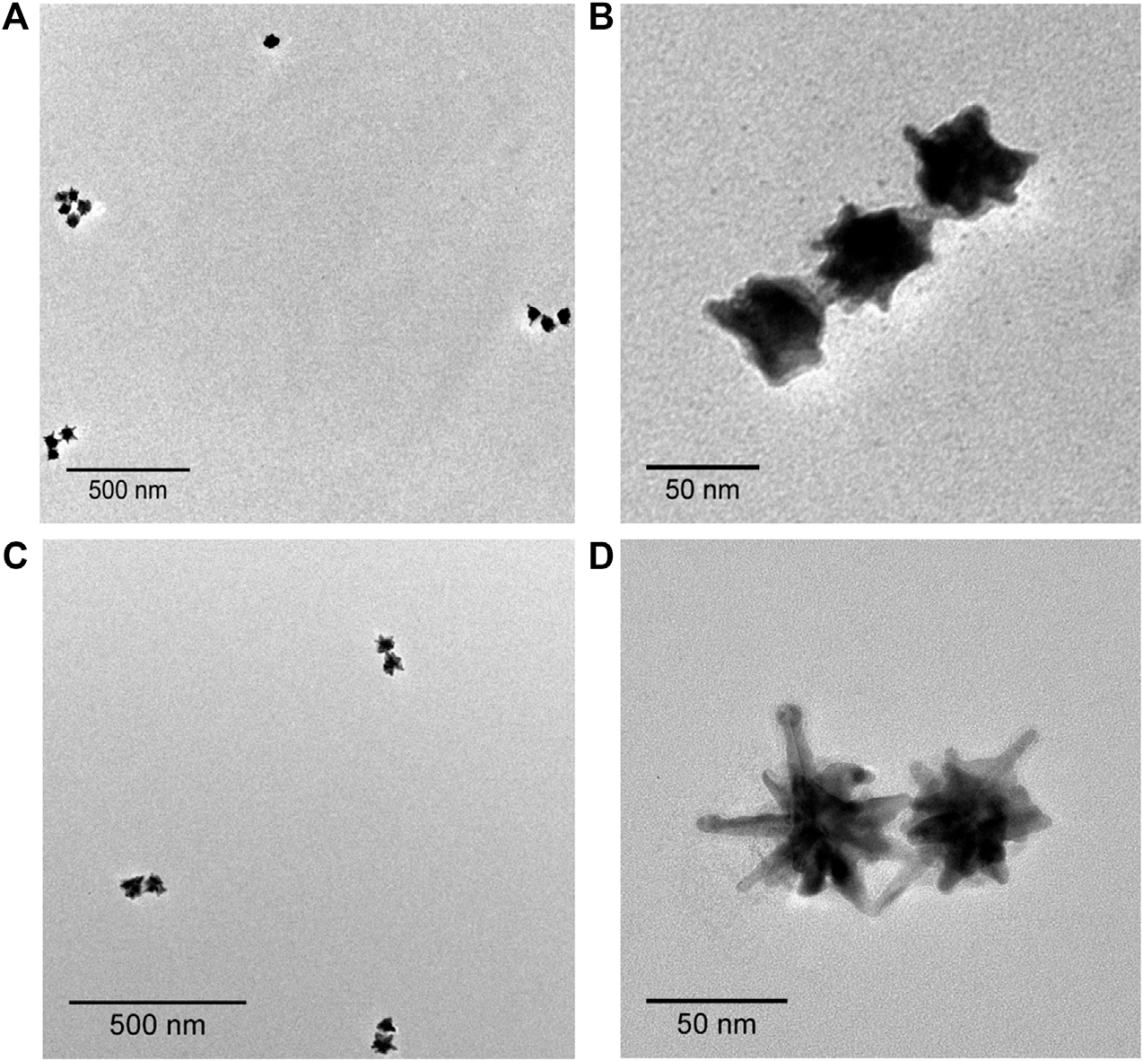
TEM images of Au@Ag NSs **(A,B)** trimer, and **(C,D)** dimer on dimerized DNA origami.

To design nanoantenna assemblies, Au@Ag NSs were selectively assembled on dimerized DNA origami via hybridization between Au@Ag NSs prefunctionalized with DNA oligonucleotides and complementary dangling staples from DNA origami (Scheme 1). The synthesized Au@Ag NSs were functionalized with thiolated DNA as explained in our previous report (Kaur et al., 2021). Supplementary Figure S2A depicts a plasmonic shift of ∼7 nm observed in DNA functionalized Au@Ag NSs. After salt stability test of DNA functionalized Au@Ag NSs (Supplementary Figure S2B), we modified the anchoring points of DNA origami dimer to form dimer and trimer assemblies. Supplementary Figure S3 depicts the AFM images of the synthesized DNA origami monomer and dimer. A set of five capturing staples on each DNA origami monomer was chosen to prepare Au@Ag NSs dimer. The Au@Ag NSs trimers were assembled on dimerized DNA origami using eight capturing staples on both origami monomers. The details of the modified staple position is illustrated in Supplementary Table S1 and Supplementary Figure S4. Supplementary Figure S5 depicts the low-resolution AFM images of Au@Ag NSs dimeric structures after immobilization on dimerized DNA origami. The height profile indicated the successful assembly of designed nanostructures on DNA origami. TEM images confirmed the assembly of Au@Ag NSs in trimeric and dimeric configuration on dimerized DNA origami as shown in Figure 2. The TEM analysis further depicted no alteration in the tips and coating of Au@Ag NSs after immobilization on the DNA origami. The interparticle gap, i.e., core–core gap in the case of dimeric assemblies after statistical analysis of 20 different TEM images was found to be 5 ± 1 nm. From the TEM images, the interparticle gap between the trimeric structures were found to be in the range of ∼1.5 nm ≤ d ≤ 7.5 nm. After considering several TEM images, the calculated yield of Au@Ag NSs dimer was around ∼64%, and Au@Ag NSs trimer were formed with yield ∼58%. The calculated yield for Au@Ag NSs dimeric structures was consistent with our previous report (Kaur et al., 2021).

### Surface-Enhanced Raman Scattering-Based Detection of Dopamine Using the Designed Nanoassemblies

**FIGURE 3:**
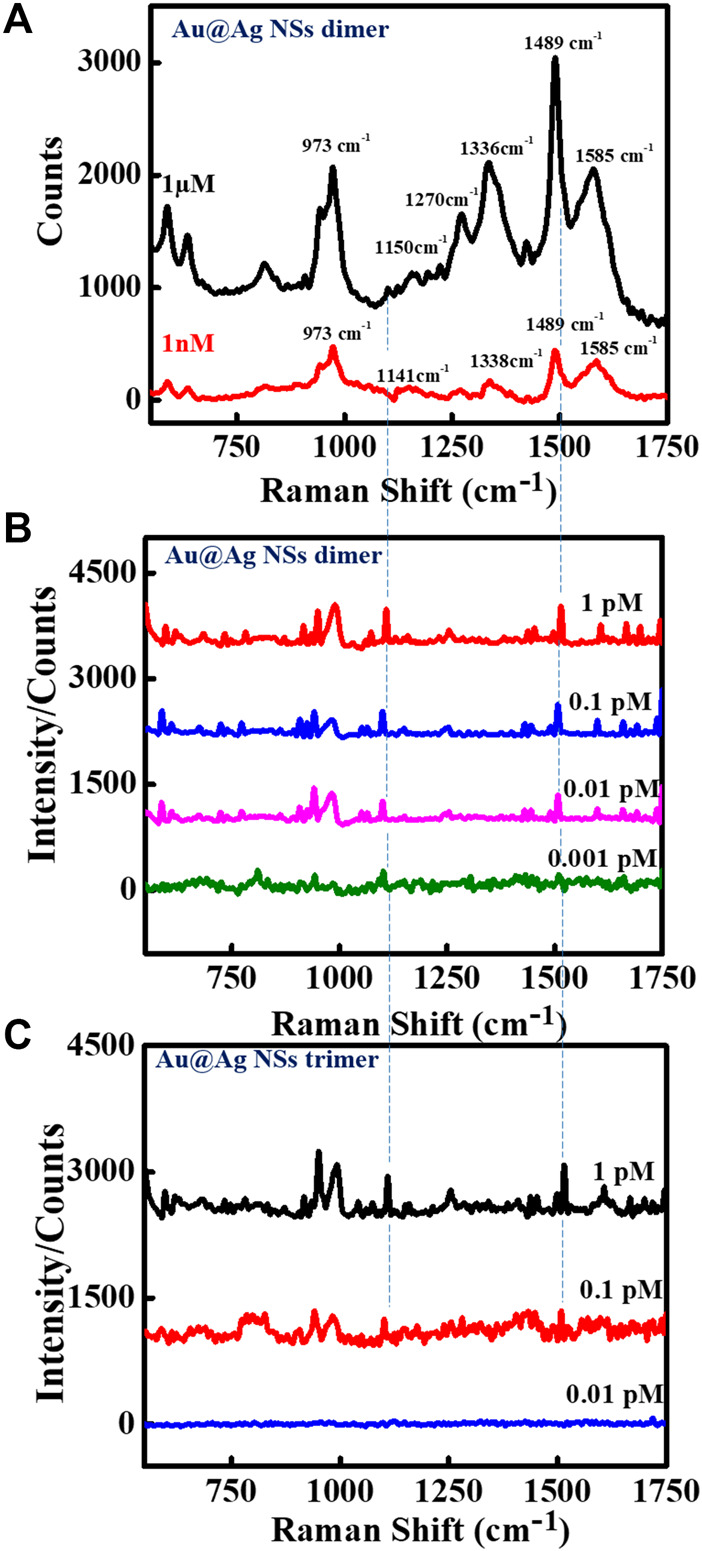
Surface-enhanced Raman scattering (SERS) spectra of Au@Ag NSs dimer with **(A)** 1 μM and 1 nM concentrations, and **(B)** pM concentration range of dopamine, and **(C)** concentration-dependent SERS spectra of Au@Ag NSs trimers with pM concentrations of dopamine.

**FIGURE 4:**
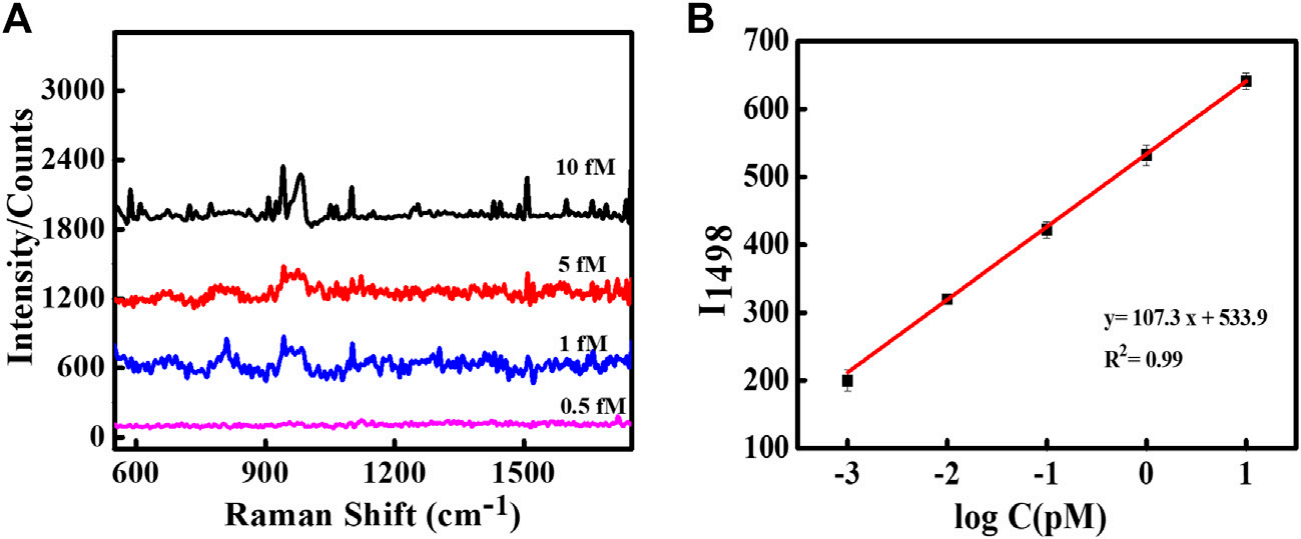
**(A)** SERS spectra of dopamine using Au@Ag NS dimer at fM concentrations. **(B)** Linear fit curve of the concentration-dependent SERS intensity of dopamine at 1,498 cm^−1^.

The sharp-edged tips of Au NSs and coating of Ag over Au NSs are expected to exclusively produce strongly localized plasmonic fields resulting in much stronger near-field enhancements. To explore this aspect, we demonstrated SERS detection of important biomarker, dopamine using designed nanoassemblies. SERS measurements were obtained by first assembling plasmonic Au@Ag nanostructures on Si substrate and recorded using 514 nm laser at 10 s acquisition time. The measurements were conducted at low laser power so as to avoid sample degradation. A known concentration of dopamine solution was drop casted on Si wafer pre-immobilized with Au@Ag NSs dimer structures. Figure 3A depicts the SERS spectra of 1 μM and 1 nM dopamine solution on Au@Ag NSs dimeric assemblies. The peaks of dopamine were obtained at 1,150, 1,270, 1,336, 1,489, and 1,585 cm^−1^, which correlated well with the existing literature. The peak at 1,489 cm^−1^ corresponds to ring stretching vibration of C–C to which oxygen is attached (Phung et al., 2018). The peaks at 1,270 and 1,336 cm^−1^ are assigned to stretching of catechol carbon-oxygen bond (Kaya and Volkan, 2012; Qin et al., 2015). The summary of peak assignment is given in Supplementary Table S2. Figures 3B, C show the baseline corrected concentration-dependent SERS spectra of dopamine with Au@Ag NSs dimers and trimers, respectively, at pM concentration range. It can be observed from the concentration-dependent plot that SERS signal of dopamine was obtained until the concentration of 0.1 pM using Au@Ag NSs trimer and ultra-low concentration of 1 fM with Au@Ag NSs dimer where the dominant peaks are varying linearly with change in concentration of dopamine. Further lowering the concentration (0.5 fM) showed no significant peaks of dopamine as depicted in the fM concentration plot of dopamine (Figure 4A).

There have been reports in the past where trimer nanostructures were able to achieve many fold increase in SERS signal enhancements. Wu et al. demonstrated the assembly of Ag NP trimers as SERS substrate for the sub-attomolar detection of a cancer biomarker, alpha-fetoprotein (Wu et al., 2015). In another study, Sergiienko et al. determined the overall increase in the SERS EFs upon dimerization and trimerization using Au and Ag NPs modified with Raman reporters (Sergiienko et al., 2017). The complex geometry and angular orientation-dependent hotspot formation in the case of trimer nanostructures create non-uniformity in signal enhancements. In a report, Pal et al. systematically investigated the effect of varying the interparticle distances and angular orientations of the size-selective Au NP dimers and trimers when the particle size is very large compared with the interparticle distance (Pal et al., 2019). They revealed that spectral features were very sensitive to the relative orientations and interparticle gap in the trimers, with even minute alterations in angular orientation of trimeric nanostructures altering the direction of light propagation. The electromagnetic enhancements increased with increase in orientation angles (θ = 30°–90°) with maximum enhancement achieved at 90° orientation. In another study, Lee et al. studied the spectral redistributions of the plasmonic modes of ∼1 nm gap trimeric Au nanostructures with varying symmetries (Lee et al., 2015). They found the increase in SERS signal from acute-angled triangles to linear triangle configuration. It can be seen from Figure 2A and Supplementary Figure S6 that the presence of different angular configurations of triangular structures could be responsible for less SERS enhancement in the case of Au@Ag NSs trimers. Along with structural complexity, in our case, the comparatively low signal enhancement in the case of trimer Au@Ag NSs can be attributed to the touching of tips in some structures due to less precise control over gap for trimers due to variation in the internal angles of the trimers. As reported previously by our group, when the core–core gap of the nanostars becomes less than ∼4 nm, while there was no gap between tips of two nanostars, reduction in electromagnetic enhancements were observed, which can be the result of quantum tunneling (Tanwar et al., 2017). However, in the case of dimeric structures, we were able to precisely control the interparticle gap, which generated higher field enhancements achieving ultra-low detection limit.

Furthermore, we explored if the SERS peaks correspond to the dopamine molecule only by recording the reference spectrum of dopamine solution with 50-nm Au nanoparticles deposited on Si substrate. As depicted in Supplementary Figure S7, the peaks matched well with the obtained SERS peaks of dopamine, which ascertained that the Raman peaks correspond to the dopamine molecule.

To ascertain that the ultra-low detection limit of dopamine is achieved due to the plasmonic effect of Au@Ag NSs assemblies, we conducted the control experiments of the system. The measurements were recorded using the low-concentration solutions of dopamine on Si substrate in the absence of Au@Ag NSs structures. It can be seen from Supplementary Figure S8 that no significant peak is observed at all the concentrations confirming the significant role of the electromagnetic enhancement generated by the designed Au@Ag NS dimer SERS platform.

We compared the SERS signal enhancement obtained due to dimeric Au@Ag NSs with the Au NS dimer on dimerized DNA origami using the same concentration (1 μM). As seen in Supplementary Figure S9, the peak intensity obtained using Au NS dimer was approximately five fold less than the SERS signal intensity of Au@Ag NSs. This clearly demonstrates that the coating of Ag on Au NSs provides enormous enhancement at the interparticle gap of the assemblies leading to significant improvement in the detection limit of dopamine. Previously, there have been many reports on maximizing the SERS enhancement due to the deposition of Ag on Au nanoparticles (Pande et al., 2007; Fernanda Cardinal et al., 2010; Tang et al., 2015; Prinz et al., 2016). In a report by Fernanda Cardinal et al., modulation of localized surface plasmons on gold nanodumbells through silver coating resulting in higher SERS enhancement was demonstrated (Fernanda Cardinal et al., 2010). In another report, Prinz et al. demonstrated increased SERS sensitivity provided by Au–Ag-core–shell NPs arranged on DNA origami substrates for single molecule detection(Prinz et al., 2016). Tang et al. demonstrated significant enhancement in electric field by deposition of Ag shell on Au NR dimers leading to ultrasensitive SERS-based detection of dopamine (Tang et al., 2015). Furthermore, it was observed by Pande et al. that better SERS signal enhancement of the probe molecule was achieved with Au_core_–Ag_shell_ particles than with bimetallic Ag_core_–Au_shell_ or monometallic gold and silver particles (Pande et al., 2007).

Thus, Ag coating on Au nanostars is expected to highly enhance the electromagnetic enhancement resulting in better SERS sensitivity. This was effectively demonstrated in our recently published report depicting the increase in electric field at tips of Au@Ag NSs compared with pure Au NSs through three-dimensional finite-difference time domain (FDTD) modeling (Kaur et al., 2021). Furthermore, we conducted the SERS measurements of different concentrations of dopamine solution with Au@Ag NSs of variable Ag thicknesses. Au@Ag NSs were prepared with average Ag shell thickness of 1.2 ± 0.5 nm (lesser coating) and 8 ± 0.7 nm (more coating) as confirmed by TEM images. Then as-prepared Au@Ag NSs were immobilized on dimerized DNA origami to prepare dimeric structures using the same set of capture strands (Supplementary Figure S10). Next, we conducted the concentration-dependent SERS measurements of the designed dimeric structures. However, no significant peaks of dopamine were obtained for 1 fM concentration in case of less (1.2-nm-thick shell) and more (8-nm-thick shell) Ag coating (Supplementary Figures S11A, B). It can be seen from Supplementary Figure S11C that the SERS intensity increased with the increase in Ag coating thickness from 1.2 to 2.4 nm. In the case of Au@Ag NSs with an average thickness of 1.2 nm, it can be observed from TEM images (Supplementary Figure S11A) that the Ag layer was deposited only on the core of the nanostars leaving the tips with no Ag coating. Hence, the low SERS sensitivity in this case may be due to the fact that Ag coating was not present uniformly on the tips of the nanostars. For Au@Ag NSs with 8 nm average thickness, the tips were found to be completely encapsulated by Ag coating, which can also lead to diminished SERS activity in this case. This can be attributed to the fact that with more coating, the sharp tips are getting diminished due to complete coverage by Ag layer, resulting in almost spherical core–shell like structures. Hence, the SERS efficiency of Au@Ag NSs with variable Ag thickness was compared, and we found maximum SERS enhancement with an optimum Ag thickness of ∼2.4 nm, which is consistent with a previous report (Feng et al., 2019). As reported by Feng et al., after comparing a series of Au@Ag core–shell NPs with different Ag thicknesses, an optimum SERS enhancement was obtained with Ag shell thickness of 2.4 nm. The Feng group stated that a thinner Ag shell is more active to prompt the electron transfer at the interface of Au and Ag compared with a thicker shell. The transferred electrons are more sufficient to compensate the thinner Ag shell that has less Ag species compared with the thicker one with more Ag species, leading to higher electron compensation. Hence, the SERS enhancement in the case of intermediate Ag coating is more compared with very less or more coating in the case of bimetallic nanostructures.

Next, we recorded the SERS spectra of Au@Ag NS monomer on dimerized DNA origami for 1 nM, 1 pM, and 1 fM concentrations of dopamine solution. The TEM image of the Au@Ag NS monomer is depicted in Supplementary Figure S12A. It can be seen from the SERS spectra (Supplementary Figure S12B) that no significant peaks in the case of pM and fM concentrations and very less intense peaks in the case of 1 nM dopamine concentration were obtained. This clearly demonstrated the highly intense electromagnetic field due to Au@Ag NSs dimeric structures leading to ultra-high sensitivity in dopamine detection.

The peak intensity at 1,498 cm^−1^ observed at concentration-dependent SERS plot of Au@Ag NSs dimer was used to calculate the limit of the detection (LOD) of the system. Figure 4B represents the linear fit curve of the concentration (log value)-dependent SERS intensity of dopamine at picomolar concentrations using Au@Ag NSs dimer at 1,498 cm^−1^. Each point in the curve represents the average value of three repeated measurements with the error bar representing their standard deviation.

The LOD was calculated to be 0.225 fM from the regression curve using the following equation (Shrivastava and Gupta, 2011):

LOD =3σ/S (1)

where σ represents the standard deviation of the blank signal, and S depicts the slope of the linear curve.

The site-specific immobilization of the Au@Ag NSs dimer on the DNA origami resulted in huge electromagnetic enhancements achieving highly sensitive, label-free detection of dopamine. Previously, Tang et al. demonstrated ultrasensitive dopamine detection using Au@Ag nanorod dimer at 6 fM concentration (Tang et al., 2015), and Zhang et al. used 3-mercaptophenylboronic acid (3-MPBA)-functionalized Ag nanoparticles to achieve dopamine detection with an LOD of 0.3 pM (Zhang et al., 2018). However, these systems used Raman reporter molecule for the indirect detection of the dopamine molecule. In another study, Wang et al. used lithographically patterned graphene-Au nanopyramid heterostructure platform for dopamine sensing at 10^–10^ level (Wang et al., 2015). Table 1 presents the comparative analysis of dopamine detection using different methods. In our case, we were able to significantly enhance the detection limit of dopamine using a biocompatible, cost-effective route. Such programmed platforms represent a unique possibility for designing robust biosensing systems having significant potential in various diagnostic applications.

**TABLE 1:** Comparison of methods of dopamine detection.

Material used	Method	LOD	Reference
β-Cyclodextrin-functionalized gold nanoclusters	Fluorescence detection	2 × 10^6^ fM	Ban et al. (2015)
BSA-Au nanoclusters	Fluorescence detection	6.2 × 10^5^ fM	Govindaraju et al. (2017)
Photoluminescent Si NPs	Fluorescence detection	3 × 10^5^ fM	Zhang et al. (2015)
Manganese-doped MoS_2_	Electrochemical impedance spectroscopy (EIS) and cyclic voltammetry (CV)	50,000 fM	Lei et al. (2020)
Carbon nanopipets	CV	10^5^ fM	Hu et al. (2019)
Gold nanostar-modified pencil graphite electrode	EIS and CV	1,500 fM	Talemi et al. (2017)
Single-walled carbon nanotubes (SWNTs)	Flow injection analysis (FIA)-based electrochemical detection	∼5 pM in 50 μl (∼0.25 fM)	Sansuk et al. (2013)
Self-assembled Au nanoparticles	SERS	5.2 × 10^6^ fM	An et al. (2011)
Graphene-Au nanopyramid hybrid platform	SERS	10^5^ fM	Wang et al. (2015)
Dual-recognition-based SERS assay	SERS	300 fM	Zhang et al. (2018)
Au/Ag nanocluster substrate	SERS	100 fM	Phung et al. (2018)
Silver nanocubes	SERS	40 fM	Lu et al. (2020)
Silver nanoparticles on the surface of metal–organic framework	SERS	320 fM	Jiang et al. (2015)
Iron-nitrilotriacetic acid-attached silver nanoparticle	SERS	600 fM	Kaya and Volkan, (2012)
Worm-like silver (Ag) clusters deposited on glass substrate	SERS	8.3 × 10^6^ fM	Palanisamy et al. (2015)
Au@Ag nanorod dimers	SERS	6 fM	Tang et al. (2015)
Au@Ag NSs	SERS	0.225 fM	Present paper

### Interference Study

**FIGURE 5:**
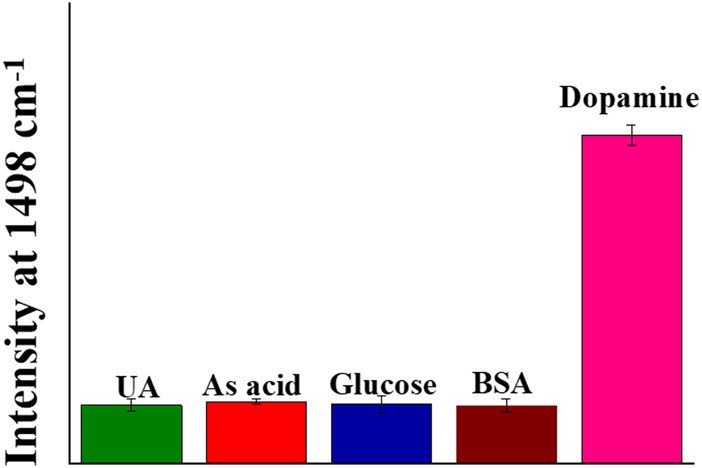
Selectivity of Au@Ag NSs assembly on DNA origami for SERS detection of dopamine.

In a biological environment, a lot of interfering substances hinder the specific detection of the analyte. Hence, to confirm if the designed system is suitable for detection in biological mediums, we test the SERS detection of four interfering molecules, i.e., uric acid, ascorbic acid, glucose, and BSA. The concentration of dopamine was 1 nM, while those of the interfering molecules was 1 μM. As depicted in Figure 5 and Supplementary Figure S13, no significant peak was obtained for all these interfering molecules. This clearly depicted weak interactions of other similar analytes with the developed SERS nanosensor in comparison with dopamine. We further conducted the Raman measurements of some structurally similar molecules, i.e., L-DOPA, L-tyrosine, and Tyramine with Au@Ag NSs dimer at 1 μM concentration. It can be seen from Supplementary Figure S14 that no significant peaks were obtained in the control sample measurements depicting highly specific detection of dopamine with the Au@Ag NSs assemblies.

The dopamine molecule can specifically get adsorbed on the surface of Ag nanoparticles. The interactions of dopamine molecule on the Ag metal surface have been previously investigated in various reports(Lee et al., 1988; Pande et al., 2009; Figueiredo et al., 2020; Rossi-Fernández et al., 2020). The adsorption of dopamine can be promoted by the catechol ring and N-protonated group as described by Figueiredo et al. (2020). In the presence of a metal surface, one of the hydroxyl group can be deprotonated and get adsorbed on the metal surface (Figueiredo et al., 2020). According to Lee et al., the SERS signal of dopamine and other catecholamines is ascribed to the formation of the metal–oxygen bond on the Ag metallic surface (Lee et al., 1988). Therefore, the designed SERS Au@Ag NSs substrate showed specific interactions in the case of dopamine molecule, while no significant SERS signal was observed in the case of other interfering molecules.

## Conclusion

In summary, we have demonstrated the design of DNA origami-assisted bimetallic Au@Ag NSs assembly for SERS-based detection of an important neurotransmitter, dopamine. Bimetallic Au@Ag NSs prepared by simple Ag deposition technique exhibited fascinating plasmon tunability and well-defined control over overall morphology of the nanostructures. The programmable arrangement of Au@Ag NSs was achieved by preparing dimers and trimers using a DNA origami template. Huge field enhancements are expected at the plasmonic hotspots formed at the junction of dimeric and trimeric assemblies enabling those structures to achieve ultra-low detection limits. The resulting dimeric assembly was able to detect dopamine with an ultra-low limit of detection of 0.225 fM. It is expected that such assemblies might open up a horizon in the biomolecule detection of analytes with ultra-high sensitivity even up to a single-molecule level.

## Data Availability

The original contributions presented in the study are included in the article/Supplementary Material. Further inquiries can be directed to the corresponding author.
