# Supplementary material for: DNA Origami-Templated Bimetallic Nanostar Assemblies for Ultra-Sensitive Detection of Dopamine
Source: Front Chem. 2021 Dec 23;9:772267. doi: 10.3389/fchem.2021.772267 (PMC8733555; doi:10.3389/fchem.2021.772267)
Supplement: Supplementary file 1 [file DataSheet1.docx]

Supplementary Material


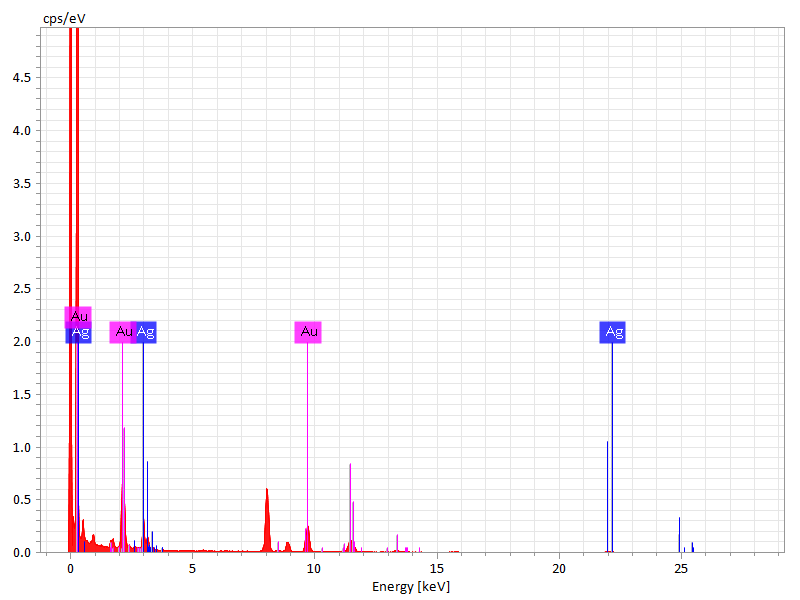


**Figure S1.** EDX spectrum of Au@Ag NS

**Figure S2.** (a) Overlapping UV-Vis spectra of Au@Ag NSs and DNA functionalized Au@Ag NSs, and (b) pictorial depiction of Au@Ag NSs (without DNA functionalization) in (i) absence, and (ii) presence of 500 mM NaCl in PBS, and (iii) DNA functionalized Au@Ag NSs in presence of 500 mM NaCl in PBS.

**Figure S3.** AFM images of DNA origami (a) monomer, and (b) dimer with height profile in inset.

**Table S1.** Position of capture staples on DNA origami

| Configuration | Monomer A | Monomer B |
| --- | --- | --- |
| Dimer | 82,83,111,85,86 | 81,82,83,111,85 |
| Trimer | 80,81,82,83,111,85,86,87 | 80,81,82,83,111,85,86,87 |

**Figure S4.** Schematic representation of staple strands on dimerized DNA origami for (a) dimer, and (b) trimer structures.

**Figure S5.** (a) AFM images of Au@Ag NSs on dimerized DNA origami, and (b) Height profile of dimer structure.

**Table S2.** Peak assignment in Dopamine SERS spectra

| **Peak range (cm^-1^)** | **Assignment** |
| --- | --- |
| 1585 | C-N stretching |
| 1489 | Ring vibration of C-C to which O is attached |
| 1336 | Catechol C-O stretching |
| 1270 | Catechol C-O stretching |
| 1148 | C-N stretching |

**Figure S6.** TEM images of Au@Ag NSs trimer structures.

**Figure S7.** Reference Raman spectrum of 1 mM dopamine solution using 50 nm spherical Au nanoparticles.

**Figure S8.** Raman spectra of different concentrations of dopamine solutions on Si wafer.

**Figure S9.** Overlay SERS spectra of 1 µM dopamine solution using Au NSs and Au@Ag NSs dimer assembled on DNA origami (5 nm gap).

**Figure S10.** TEM images of (a) Au@Ag NSs, and (b) Au@Ag NSs dimer with average Ag thickness of 1.2 nm, and (c) Au@Ag NSs, and (d) Au@Ag NSs dimer with average Ag thickness of 8 nm.

**Figure S 11.** Concentration dependent SERS measurements of dopamine solution using Au@Ag NSs dimer with average Ag thickness of (a) 1.2 nm, and (b) 8 nm, and (c) overlay SERS spectra of (i) Au@Ag NSs monomer, and Au@Ag NSs dimer with (ii) 1.2 nm, (iii) 8 nm, and (iv) 2.4 nm Ag thickness at 1 pM dopamine concentration.

**Figure S12.** (a) TEM image of Au@Ag NS monomer on dimerized DNA origami, and (b) Concentration dependent SERS spectra of dopamine using Au@Ag NSs monomer.

**Figure S13.** Overlay Raman spectra of different interferring chemicals (1µM) and dopamine solution (1 nM) using Au@Ag NSs dimer structures.

**Figure S14.** Overlay Raman spectra of different structurally similar molecules of dopamine (1 µM solution) using Au@Ag NSs dimer structures.
